# Assessing and improving the reliability of meta-analyses of hazard ratios derived from published time-to-event data

**DOI:** 10.1186/1745-6215-14-S1-O93

**Published:** 2013-11-29

**Authors:** Jayne Tierney, David Fisher, Sarah Burdett, Lesley Stewart, Mahesh Parmar

**Affiliations:** 1 MRC Clinical Trials Unit Hub for Trials Methodology Research, London, UK; 2 Centre for Reviews and Dissemination, York, UK

## Background

Trial design and conduct is informed by systematic reviews and meta-analyses (MAs). For time-to-event outcomes, they are typically based on hazard ratios (HRs). Ideally, these are calculated from individual patient data (IPD). Alternatively, they can be extracted from published aggregate data (AD) or estimated from other statistics or from Kaplan-Meier (KM) curves. As reporting and the reliability of each estimation method is likely variable, we investigate how HRs from AD and IPD compare.

## Methods

We used 18 of our published IPD systematic reviews and MAs. For our primary outcome of overall survival, we extracted or estimated logHRs and standard errors (SEs) from eligible trials wherever possible. We compared HRs from AD and IPD at the trial and meta-analysis level, using Bland-Altman methods to assess agreement.

## Results

Of 245 unique trials eligible for the meta-analyses, HRs were available from IPD for 199 (81%), and from AD for 155 (63%) including 75 (48%) based on KM curves. Trial logHRs from AD were biased towards benefit compared to IPD (5% on HR scale, p=0.009), and SEs were inflated (+0.23 SDs, p<0.001). Bias was not influenced by estimation method (p=0.84). Similar bias was seen in MA logHRs from AD (3.5% on HR scale, p=0.031), and again inflated SEs (+0.87 SDs, p<0.001). The level of bias varied by meta-analysis.

## Conclusions

Although MAs of AD-derived HRs can be biased, their use is widespread and needed when the IPD approach is infeasible. We will present results on causes of these biases and guidance on overcoming them.

